# The impact of typological similarities and differences between German and Italian on the acquisition of language-specific phonetic cues in bilingual children: insights from the T-complex

**DOI:** 10.3389/fnhum.2024.1482052

**Published:** 2024-12-23

**Authors:** Theresa Bloder, Yasuaki Shinohara, Tanja Rinker, Valerie L. Shafer

**Affiliations:** ^1^Department of Languages and Literatures, Catholic University Eichstätt-Ingolstadt, Eichstätt, Germany; ^2^Faculty of Commerce, Waseda University, Tokyo, Japan; ^3^Ph.D. Program in Speech-Language-Hearing Sciences, The Graduate Center, The City University of New York Graduate Center, New York, NY, United States

**Keywords:** bilingualism, language development, electrophysiology, auditory evoked potentials, T-complex, speech sound processing

## Abstract

**Introduction:**

Lateral temporal neural measures (Na and T-complex Ta and Tb) of the auditory evoked potential (AEP) index auditory/speech processing and have been observed in children and adults. While Na is already present in children under 4 years of age, Ta emerges from 4 years of age, and Tb appears even later. The T-complex has been found to be sensitive to language experience in Spanish-English and Turkish-German children and adults. In particular, Ta elicited to a vowel has been found to be sensitive to language experience in bilingual preschool children. This paper examines neural responses in 4-to-6-year-old Italian-German bilingual and German monolingual children using language-specific phonetic cues for voicing.

**Methods:**

We tested children's processing of voicing features in bilabial stop consonants in relation to (1) their language status (i.e., being monolingual vs. bilingual) as well as to (2) their relative amount of current exposure to the heritage (Italian) and the societal language (German). Italian-German bilingual and German monolingual children were hypothesized to encode the temporal properties of a set of Voice Onset Time (VOT) stimuli differently as indexed by Ta and Tb.

**Results:**

The results revealed no main effects of language group, but interactions of group with hemisphere and stimulus. In particular, bilingual children showed less hemispheric differentiation and an attenuated (less positive) response at the right site (T8) for the 0 ms VOT stimulus during the Ta-Tb time window. Children with more German (and consequently, less Italian) input showed a more positive T8 response for the Na, Ta and Tb time intervals.

**Discussion:**

These findings partially replicated previous studies, but also revealed that stimulus factors modulate the response. They suggest that a delay in commitment is found only in bilinguals with less input in the target language, and those who are strongly dominant in one of the two languages will resemble monolinguals in the development of T-complex responses. However, the finding of greater Na positivity for German-dominant bilinguals suggests that their specific experience also influences processing, but perhaps via a different mechanism than found for the more balanced bilinguals.

## 1 Introduction

Establishing a phonological system lays a crucial foundation for subsequent language acquisition. A large amount of previous research established that newborns are inherently capable of discriminating a wide range of speech sounds across various languages. However, within the 1^st^ year of life, infants attune their perceptual ability to the specific sound patterns of their surrounding language(s) (Cheour et al., 1998; Kuhl et al., 2008, 1992). Neurobiological investigations into speech and language development demonstrate how both intrinsic and environmental factors influence the formation of a child's phonological system (Shafer et al., 2011b; Yu et al., 2019). Particularly, bilingual development, that is being exposed to two languages during initial language acquisition, has been demonstrated to impact the formation of the phonological system.

Research indicates that being exposed to a second language (L2) at an early age (i.e., before the age of 5 years) facilitates the ability to distinguish and categorize speech sounds in both languages at a native or native-like level (Bosch and Sebastián-Gallés, 2003; Flege et al., 1997; Hisagi et al., 2015). However, only a small number of studies have thoroughly explored the progression of L2 speech perception particularly in the pre-school years. The existing literature on this topic suggests that even after 2 years of exposure to the L2, differences persist in speech perception and processing compared to monolingual children (Rinker et al., 2010). Furthermore, there is notable variability in L2 phonological development in the pre-school years influenced by various factors, including input conditions, language similarity, and age of initial exposure (Carroll, 2017; Kehoe and Havy, 2019).

Neural indices of speech processing demonstrate sensitivity to distinctions in first language (L1) vs. L2 phonological processing. These indices have the capability to uncover processing variations that may not be apparent at the behavioral level (Hisagi et al., 2015). The majority of investigations on this topic involving bilingual children have been concerned with speech discrimination (e.g., Cheour et al., 2002; Rinker et al., 2010; Shafer et al., 2011a; Yu et al., 2019). Fewer studies, however, have focused on speech encoding in the brain, which can also provide valuable insights into speech sound processing (Rinker et al., 2022; Wagner et al., 2013). Measures of neural encoding include the auditory evoked potentials (AEPs) P1-N1-P2 recorded at frontocentral sites and the T-complex Ta and Tb at lateral temporal sites (Wolpaw and Penry, 1975).

The T-complex AEPs, specifically Ta and Tb, reflect essential auditory-sensory processing (Wolpaw and Penry, 1975), and are elicited in response to both speech and non-speech auditory stimuli (Bishop et al., 2012). In adults, the T-complex AEPs comprise a positive peak occurring between 105 ms and 115 ms (Ta), and a negative peak between 150 ms and 160 ms (Tb); an earlier negativity, Na peaks between 50 ms and 100 ms (Wolpaw and Penry, 1975). This Na, however, may reflect the same sources as the frontocentral P1 in superior temporal cortex, whereas Ta and Tb are argued to have sources in lateral auditory cortex (Ponton et al., 2000; Tonnquist-Uhlén, 1996; Shafer et al., 2015).

Studies of the maturation of the T-complex peaks illustrate a protracted developmental trajectory (Shafer et al., 2015), with only the Na peak being consistently present in response to a vowel stimulus in children under 4 years of age. In the Shafer et al. (2015) study, the Ta peak emerged between 4 and 8 years of age, while Tb was not readily identifiable in children's data at 7 years of age but was present in adult data.

The T-complex is also influenced by language experience (Rinker et al., 2017; Wagner et al., 2013). Rinker et al. (2017) examined monolingual and bilingual children's lateral-temporal AEPs to the /ε/ (a vowel that is more prototypical for the monolinguals' language) and found that Ta was less positive in amplitude for many of the bilinguals compared to the monolinguals. The authors proposed that limited exposure to the phonology of the L2 led to less mature patterns in the T-complex for both Spanish-English and Turkish-German bilinguals. Interestingly, in a follow-up analysis with the Turkish-German bilinguals from Rinker et al. (2017), the bilingual children showed attenuation of the Ta amplitude to a non-speech tone, as well as to the vowel stimulus (Rinker et al., 2022). Shafer (2024) suggested that bilingual children have not yet neurally committed to the selective perception routines (SPRs) of their native languages (see also Kuhl et al., 2008). Additional evidence suggests that the neural sources underlying the T-complex are important for language acquisition. Several studies have found that T-complex peaks are also attenuated in children with developmental language disorder (DLD; Shafer et al., 2011a; Bishop et al., 2012; Tonnquist-Uhlén et al., 1996). It remains unclear why the T-complex tends to be attenuated for both children with DLD and children with bilingual experience (Rinker et al., 2022). It is critical to extend research on the T-complex measures to additional language pairs in bilingual language acquisition to fully understand how language experience modulates development of neural processing of the neural sources underlying this measure.

Previous studies have generally examined the T-complex Ta and Tb at both left and right sites (e.g., Tonnquist-Uhlén, 1996; Shafer et al., 2015; Rinker et al., 2017, 2022). Historically, strong claims have been made about the special role of the left hemisphere in language (e.g., Hugdahl, 2000) and that poor processing in the left hemisphere would account for language disorders (Tonnquist-Uhlén, 1996). For these reasons, we examine whether language experience affects T-complex responses to speech sound stimuli differently in the left and right hemisphere.

## 2 The present study

The present study addresses the impact of typological similarities and differences between German and Italian on the acquisition of a language-specific phonetic cue, Voice Onset Time (VOT). VOT in many languages determines a two-way voicing distinction for stops (e.g., /b/-/p/), but the boundary placement differs with most Germanic languages (except Dutch) showing a boundary at a long-lag VOT, in which laryngeal voicing of the vowel is delayed after consonant release, whereas most Romance languages show the boundary in VOT lead, where laryngeal voicing begins during the consonant closure (Abramson and Whalen, 2017). We test how bilingual Italian-German vs. monolingual German children living in Germany process voicing features in bilabial stops and how neural processing relates to their relative amount of current exposure to the heritage (Italian) and the societal language (German). Bilabial stops (i.e., /b/ vs. /p/) were selected because the phonetic properties used to distinguish /ba/ from /pa/ differ in the two languages. Specifically, German contrasts short-lag VOT with long-lag, aspirated VOT, whereas Italian contrasts short-lag VOT with prevoiced VOT. We are particularly interested in whether different types of stimuli elicit different T-complex patterns. In particular, bilingual Italian-German children are expected to have had the greatest exposure to short-lag VOT which is present in both languages, whereas their experience with either long-lag or prevoiced VOT will depend on the input pattern of the two languages. In contrast, monolingual German children are expected to have had substantial exposure to long-lag and short-lag VOT, but no experience with prevoiced VOT.

We also examine whether experience with the language is more readily observed for contrasts that are close to the category boundary of a phonemic contrast. For example, native listeners of German (and likewise English) generally place the phoneme boundary between the short-lag and the long-lag VOT, somewhere between 20 and 30 ms (e.g., Keating et al., 1981) and have no phonemic boundary between voicing lead and short-lag VOT. Bilingual learners may place the boundary in a different location. For this reason, we included stimuli that were near the boundary (e.g., +36 ms VOT) and far from the boundary (e.g., +92 ms VOT) to examine whether bilingual experience would modulate the response to a phonetic form near a phonemic boundary. This experience will be different for monolingually- and bilingually-exposed children.

We hypothesize that bilingual children will show an attenuated Ta-Tb amplitude compared to monolingual children. This attenuation will indicate that these children have not yet neurally committed to their native-language SPRs which may be related to relatively less input in both languages. An alternative hypothesis is that the Ta-Tb amplitude is modulated only for the speech sounds that are not in the child's language. In this case, we will see attenuated Ta-Tb to the prevoiced [ba] stimuli for the German monolinguals. We also hypothesize that the group differences will be greater for the contrasts closer to the boundary (i.e., the difficult +36 ms and −36 ms VOTs), compared to easier (i.e., −112 ms and +92 ms) VOTs, because experience with stimuli close to the boundary will be dependent on the input. Specifically, a bimodal distribution is expected to be defined by a boundary (Maye et al., 2002). Na was included in the analyses to ensure that it is present in response to all stimuli. Finally, we predict that if there is an effect of hemisphere, then the right site (T8) will show a greater difference between the monolingual and bilingual participants as demonstrated in Rinker et al. (2017).

## 3 Materials and methods

### 3.1 Participants

A total of 40 children with typical language development between the ages of 47 months (3 years and 11 months) and 73 months (6 years and 1 month) participated in this study. Twenty-four of the children were simultaneous or early-sequential bilingual Italian-German speaking children (18 females) with a mean age of 59.00 months (*SD* = 8.86) and 16 were monolingual German speaking children (6 females) with a mean age of 61.06 months (*SD* = 6.42). The Wilcoxon Rank Sum Test demonstrated that children's age did not differ between the two groups (*W* = 163, *p* > 0.05). At the time of their participation in this study, all children were living and attending a kindergarten in Germany.

Twenty-two of the bilingual children were born and raised in Germany and two in Italy; those two had moved to Germany before 3 years of age. All bilingual participants had at least one native Italian-speaking parent and were exposed to Italian on a daily basis, although to varying degrees. Participants with two Italian-speaking parents (*n* = 4) had been exposed to German for a minimum of 2 years. All but one of the bilingual Italian-German children were enrolled in a bilingual Italian-German kindergarten program. This particular child was one of the four children with two Italian-speaking parents. Overall, the bilingual children's dual language environment provided them with frequent language input from multiple speakers in both Italian and German. A detailed Language Background Questionnaire (LBQ) was used to provide an objective estimate of how much each bilingual child heard and spoke each of their two languages over a typical week. The data focused on two main areas: (1) language use at home, examining the amount of Italian and German spoken by family members (e.g., caregivers, siblings) to the child (input) vs. by the child (output); and (2) language use outside the home, assessing hours spent in external environments (e.g., kindergarten, with other caregivers, in leisure activities, with friends) and how much Italian and German the child encountered and used in these settings. Caregivers rated this exposure using a seven-point scale. Based on these ratings, a composite score of each child's language exposure (input and output) was calculated, following a method similar to Cattani et al. (2014) (see Bloder et al., 2024 for more details regarding the computation of this score). Table 1 provides an overview of bilingual children's relative amount of language input and output.

**Table 1:** Overview of bilingual Italian-German speaking children's current language experience as assessed with the LBQ.

Relative amount of current language input	Italian	*M* = 43.72, *SD* = 18.33
	German	*M* = 56.28, *SD* = 18.33
Relative amount of current language output	Italian	*M* = 37.03, *SD* = 25.02
	German	*M* = 62.97, *SD* = 25.02

Measures of relative amount of language input and output are displayed in percent (%). Due to one family failing to return the completed questionnaire, *n* = 23 are available.

All monolingual German children had two monolingual German-speaking parents. They were born and raised in Germany and attended a monolingual German kindergarten program at the time of their participation in the study. They had no experience in being exposed to Italian (or any similar voicing language, such as Spanish).

Table 2 displays the results of the German language performance and nonverbal intelligence scores for both groups (monolingual vs. bilingual) assessed by the German language screening for children with German as a second language, Linguistische Sprachstandserhebung Deutsch als Zweitsprache (LiSe-DaZ; Schulz and Tracy, 2011), and the Colored Progressive Matrices (CPM; Bulheller and Häcker, 2001), respectively. According to the Shapiro-Wilk normality test, the scores were not normally distributed in either of the two groups; therefore, Wilcoxon Rank Sum Tests were conducted to compare the scores between groups. Except for the verb placement test, bilingual and monolingual participants did not differ in their German language performance or non-verbal intelligence scores. It should be noted that all participants in the German monolingual group marked the maximum score of 4 in the verb placement test.

**Table 2:** German language performance and non-verbal intelligence scores assessed with the LiSe-DaZ and the CPM per group (German monolinguals vs. Italian-German bilinguals).

		**German monolinguals (*n* = 16)**	**Italian-German bilinguals (*n* = 24)**	**The Wilcoxon Rank Sum Test**
LiSe-DaZ	Verb placement	*M* = 4.00, *SD* = 0	*M* = 3.71*, SD* = 0.46	*W* = 136, *p* = 0.020
	Subject-verb-agreement	*M* = 3.56*, SD* = 0.96	*M* = 3.54*, SD* = 0.98	*W* = 188.5, *p* > 0.05
	Word classes	*M* = 51.76*, SD* = 3.71	*M* = 51.84*, SD* = 8.77	*W* = 229.5, *p* > 0.05
	Case markings	*M* = 56.81*, SD* = 10.12	*M* = 55.29*, SD* = 14.12	*W* = 192.5, *p* > 0.05
CPM	Raw scores	*M* = 16.56, *SD* = 3.42	*M* = 16.17, *SD* = 5.33	*W* = 156, *p* > 0.05

For the LiSe-DaZ subtests (i.e., verb placement and subject-verb-agreement) the maximum score is 4; for the subtests of word classes and case markings T-scores are displayed, where the standardized mean is 50 and SD is 10. For each assessment, the Wilcoxon Rank Sum Test was conducted to compare the score difference between groups.

### 3.2 Stimuli

**Figure 1:**
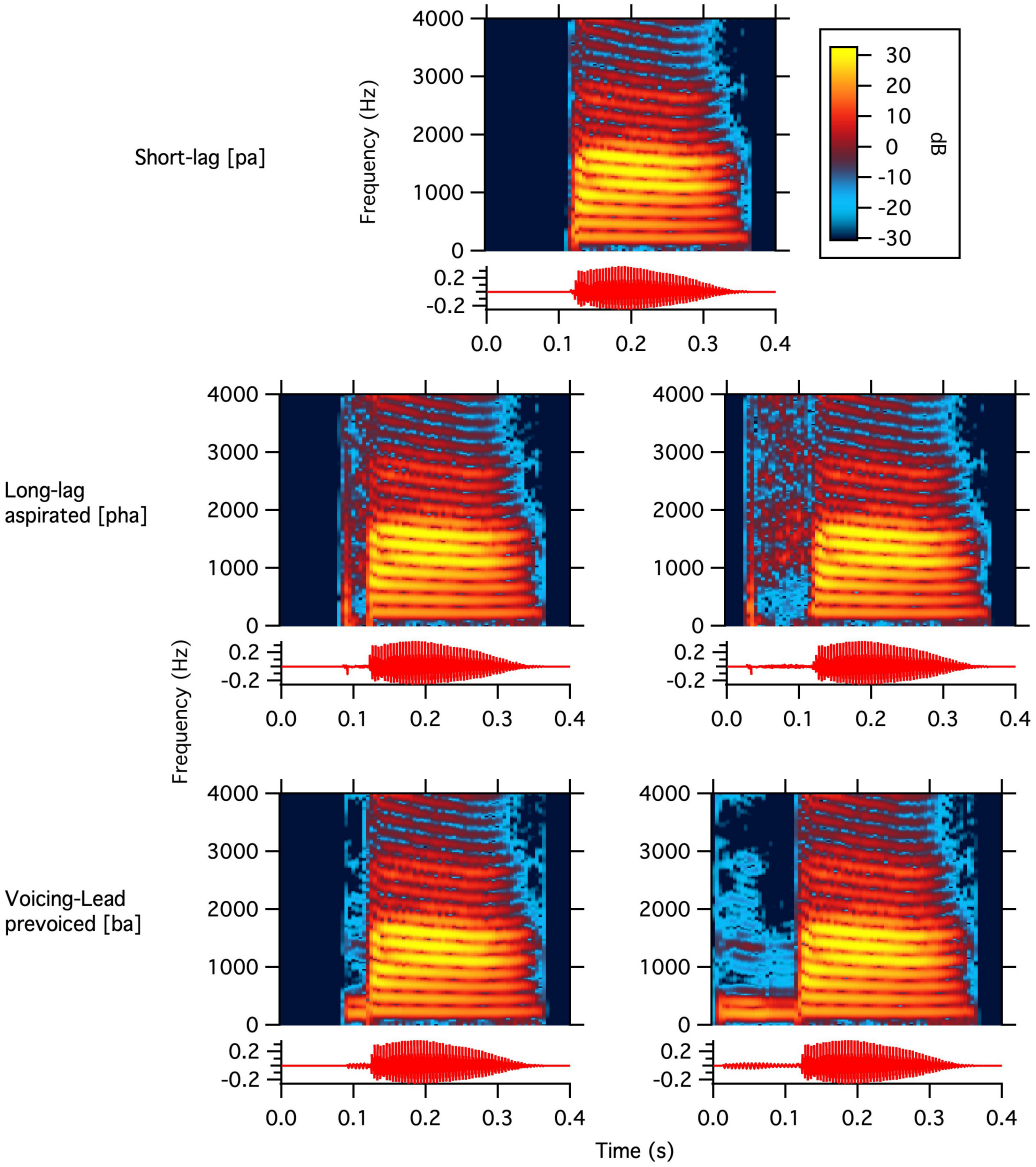
Waveforms and narrowband spectrograms of the five bilabial stimuli. The top graph shows 0 ms VOT, the middle two graphs show +36 ms **(left)** and +92 ms VOT **(right)**, and the bottom two graphs show −36 ms **(left)** and −112 ms VOT **(right)**. The color scale shows amplitude on a linear scale.

Natural speech stimuli were recorded by a native speaker of Bengali because the language uses both voicing and glottal laryngeal properties (described as the features of spread glottis for aspiration/breathiness and voice for the onset of vocal fold vibration relative to stop release). These are long-lag aspirated [p^h^a] = [+spread glottis][-voice], short-lag unaspirated [pa] = [–spread glottis][–voice], and prevoiced [ba] = [–spread glottis][+voice]. We chose a Bengali speaker to avoid a bias toward German or Italian and because this allows equally natural-sounding stimuli at both ends of the voicing continuum. The stimuli were recorded in a sound-shielded booth; the speaker was instructed to produce the syllables [ba], [pa], and [p^h^a] in isolation. The open central vowel /a/ was chosen because it is articulatorily similar in Bengali, Italian, and German (ud Dowla Khan, 2010; Rogers and d'Arcangeli, 2004; Kohler, 1990). After recording, the stimuli were edited in Praat (Boersma and Weenink, 2018) such that they would all include the same vowel portion (see Figure 1). To this end, the recordings were segmented into their consonant and vowel components, and then recombined to ensure that each stimulus contained an identical vowel segment. This manipulation allowed any observed differences in participants' brain responses to be attributed exclusively to variations in VOT. Along the same lines, VOT duration was manipulated (i.e., extended or shortened) to obtain an “easy” (i.e., further from the native adult VOT boundary) and a “difficult” (i.e., closer to the native adult VOT boundary) version of both the long-lag aspirated [p^h^a] (VOT = +92 and +36 ms) and the prevoiced [ba] (VOT = −112 ms and −36 ms). We refer to [p^h^a] as German-like and [ba] as Italian-like. The short-lag [pa] has the voicing of the vowel begin immediately after the burst (VOT = 0 ms). The stimuli were selected in a behavioral ABX task, using several VOT steps on the continuum from prevoiced to aspirated, with monolingual adult speakers of German (*n* = 8) and Italian (*n* = 11), where the extreme VOTs served as A or B. Germans perceived positive VOT (VOT = +96 and +36 ms) as /pa/ and VOTs < 30 ms as /ba/ (VOT = −112 ms and −36 ms and 0 ms). In contrast, Italians perceived VOTs leading the stop burst (−112 and −36) as /ba/ and those ≥0 as /pa/ (Bloder et al., 2024).

### 3.3 Procedure

We used an oddball design to elicit the AEP measures. The paradigm was initially chosen to elicit the Mismatch Negativity (MMN), which is reported in a different paper (see Bloder et al., 2024). Eighty percent of all stimuli were the repeated 0 ms VOT [pa] standards. In the Difficult condition, two deviants (+36 ms [p^h^a] and −36 ms [ba]) were presented, each with 10% probability. Likewise, in the Easy condition, two deviants (+92 ms [p^h^a] and −112 ms [ba]) were each presented with 10% probability. The inter-stimulus interval was 722 ms from the offset of the vowel to the onset of the next vowel. As a result, the ISI between vowel offset and burst onset differed for each stimulus type (with the longer ISI for the 0 ms VOT [pa]). From these two MMN oddball paradigm conditions, only the standard 0 ms VOT was analyzed for this paper. At the end of each condition, each speech sound that had served as a deviant was repeated 100 times using the same ISI as for the Easy and Difficult oddball conditions (which we refer to as the deviant-control condition). The four VOTs in the deviant-control conditions were also analyzed for the current study. The stimuli were presented so that they were perceived as aligned according to the vowel onset rather than the onset of the aspiration or prevoicing, with the goal to present them with a sense of regular rhythm (perceived in terms of the timing of the peak amplitude of the vowel). By doing this, differences in VOT could be attributed to the onset of voicing of the consonant, rather than a difference in rhythm. No active participation was required from the children; they were allowed to watch a muted cartoon on an iPad screen, while the auditory stimuli were presented binaurally through headphones at 60 dB SPL, delivered via Eprime software (Psychology Software Tools, Pittsburgh, PA, United States).^1^ This setup served to maintain children's engagement throughout the EEG recording while at the same time drawing their attention away from the auditory stimuli to facilitate the assessment of pre-attentive processing of our stimuli.

As noted above, the current study focuses on stimulus encoding using AEP measures from temporal sites, and thus, examines brain responses to the 0 ms VOT [pa] stimulus that served as the standard in the MMN oddball paradigm conditions, and the brain responses to the four deviant stimuli when presented in the deviant-control condition (−112 ms [ba], −36 ms [ba], +36 ms [p^h^a] and +92 ms [p^h^a]). We focus on these stimuli because the goal was to examine stimulus encoding, and because the responses to the deviant stimuli in the MMN oddball paradigm conditions would have been confounded by the stimulus-change effect.

### 3.4 Recording and processing of the data

The EEG signal was recorded at a 500 Hz sampling rate using a BrainProducts Inc. EEG system via a PC laptop running BrainVision Recorder software. Online bandpass filtering was DC to 131 Hz. The system includes the LiveAmp 32 amplifier to record the continuous EEG from the scalp using 32 actiCAP slim electrodes mounted in the actiCAP snap electrode cap. Electrodes were placed over frontal (Fp1, Fp2, Fz, F3, F4, F7, F8, FT9, FT10, FC5, FC6, FC1, and FC2), central (Cz, C3, C4, CP1, CP2, CP5, and CP6), posterior (Pz, P3, P4, P7, P8, Oz, O1, and O2), and temporal sites (T7, T8, TP9, and TP10), using the 10/10 montage. Electrodes were filled with SuperVisc electrolyte gel to reduce impedances below 50 kΩ. An additional electrode placed at FCz served as the online reference during data collection. The offline analysis was conducted in BrainVision Analyzer software v2.1 (BrainProducts Inc.). After visual inspection of the raw data for each participant, channels contaminated by noise were reconstructed using triangulation and linear interpolation. The data were filtered (IIR filter, low cut-off: 0.1 Hz; high cut-off: 30 Hz, 50-Hz notch filter), and eye-blink corrected using independent components analysis (ICA). Trials with a min-max >70 μV were removed. Artifact-free EEG segments were averaged for each stimulus type separately. Averaged data were re-referenced to an average reference and then baseline-corrected (pre-stimulus baseline of 200 ms).

**Figure 2:**
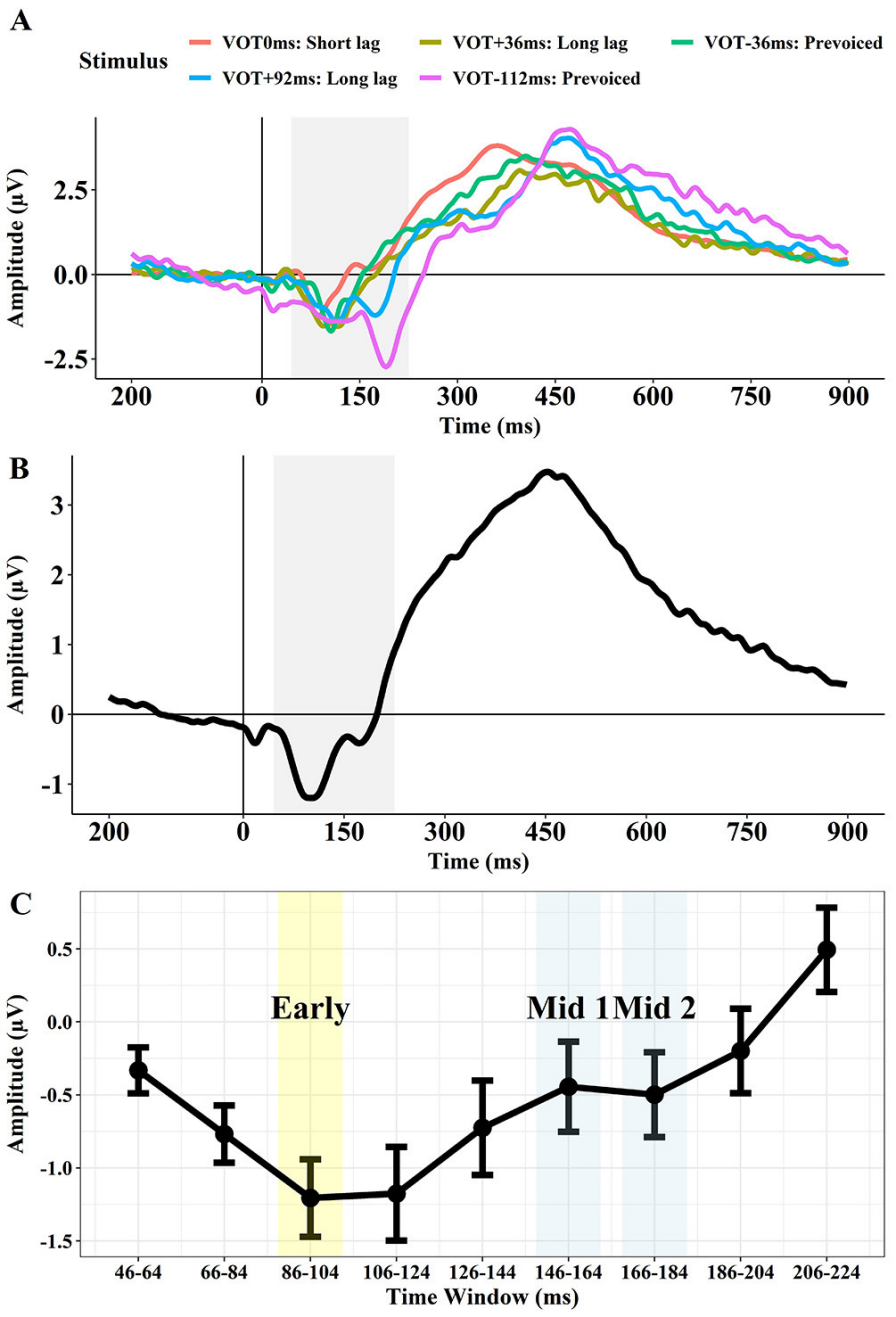
Grand mean waveforms of the AEPs for the five stimuli (VOT 0 ms, VOT +36 ms, VOT −36 ms, VOT +92 ms, VOT −112 ms) across T7 and T8 **(A)**, the averaged waveform for the five stimuli **(B)**, and the mean amplitude and confidence intervals for each of the 18 ms time windows from 46 to 224 ms **(C)**.

We selected the 86–104 ms time window as the Early interval that was supposed to reflect the Na component, the 146–164 ms window as the Mid 1 interval supposed to reflect Ta, and the 166–184 ms window as the Mid 2 interval supposed to reflect the Tb component. Figure 2 displays the grand mean waveforms of the AEPs for each stimulus (i.e., VOT 0 ms, +36 ms, −36 ms, +92 ms, and −112 ms) across the two sites (T7 and T8; Figure 2A) and the averaged waveform across the five stimuli (Figure 2B). Figure 2A shows an early negative peak, consistent with the Na, for all stimuli peaking in the 86–104 ms time window. The +92 ms and −112 ms VOT stimuli exhibit a second negative peak between 150 ms and 200 ms, which likely reflects an overlapping Na response to the onset of the vowel. Based on these observations, we created 9 time windows of 18-ms intervals from 46 ms to 224 ms after the onset of the stimulus (Figure 2C).

### 3.5 Analysis

Two linear mixed effects models were constructed. One was for the Early time interval reflecting the Na component and the other one was for the Mid 1 and 2 time intervals supposed to reflect Ta and Tb, respectively. Na was analyzed separately because it is believed to have a different cortical source than Ta and Tb (e.g., Shafer et al., 2015). The linear mixed effects model for the Early time interval included the fixed effects of Site (T7: Left vs. T8: Right), Group (monolinguals vs. bilinguals), and Stimulus (VOT: 0 ms, +36 ms, −36 ms, +92 ms, and −112 ms). All possible two-way and three-way interactions were also included, as well as by-participant random intercepts. Similarly, the linear mixed effects model for the Mid 1 and 2 time interval included the fixed effects of Site, Group, Stimulus, and Time interval (Mid 1 vs. Mid 2). The six 2-way interactions (Group & Time interval, Group & Stimulus, Time interval & Stimulus, Group & Site, Time interval & Site, and Stimulus & Site) and a 3-way interaction (Group, Stimulus & Site) were also included as well as by-participant random intercepts. Other factors that did not improve the model fit were excluded during the model comparison process according to the Akaike Information Criterion (Barr et al., 2013; Bates et al., 2015; Matuschek et al., 2017). For both models, orthogonal contrasts were used for all categorical variables. *Post hoc* analyses were conducted for each model, using the emmeans function in R (Lenth, 2024). The *p*-values were adjusted with the Tukey method, when there were multiple comparisons within a variable.

## 4 Results

**Figure 3:**
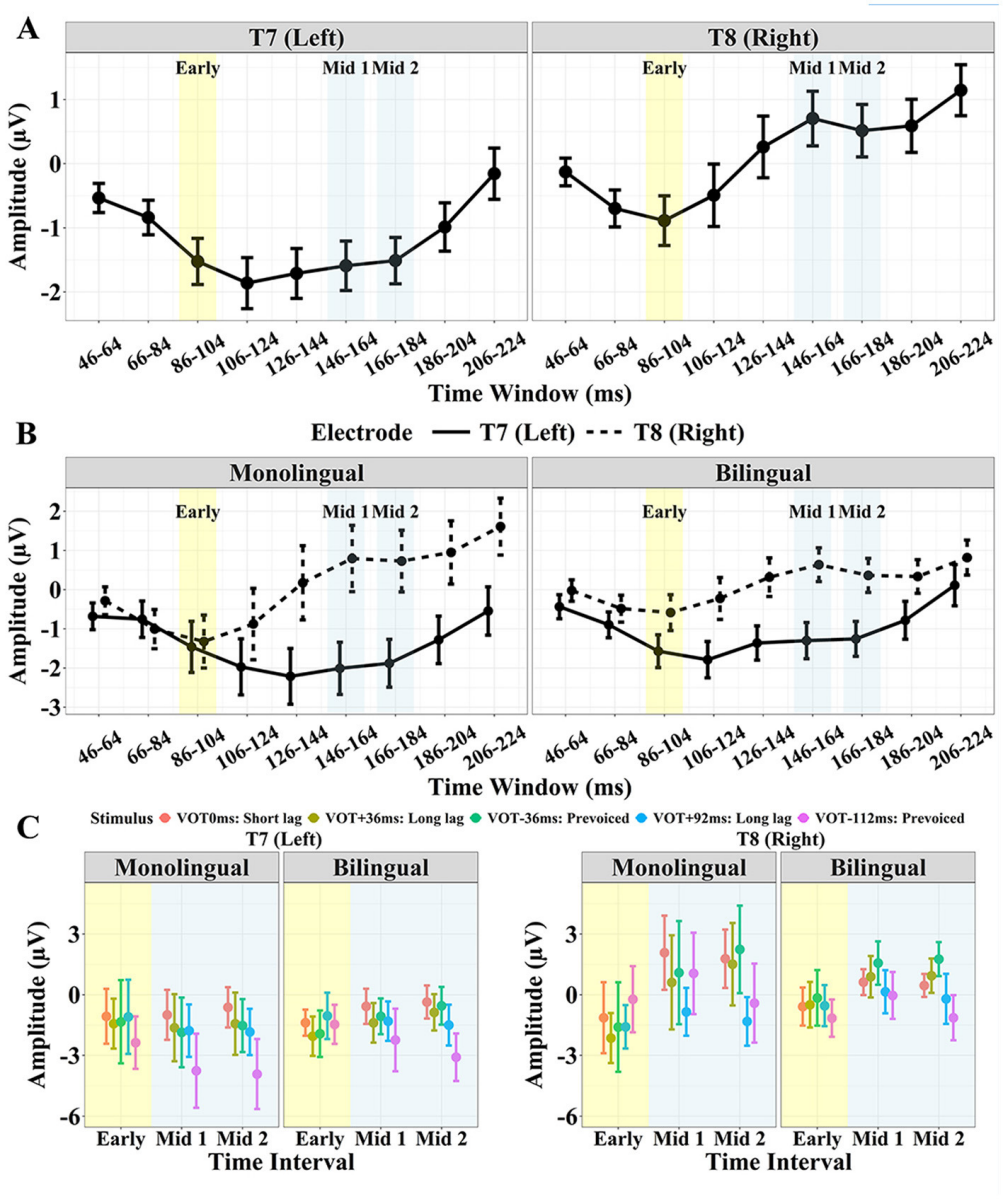
AEP mean amplitude and confidence intervals (CIs) for T7 and T8; **(A)** averaged across stimulus and group for each of the 18 ms time windows; **(B)** Monolingual vs. Bilingual group averaged across stimulus for each time window; **(C)** Monolingual vs. Bilingual group for each stimulus for the three target time intervals (Early: 86–104 ms, Mid 1: 146–164 ms, and Mid 2: 166–184 ms). The stimulus VOTs from left to right are 0 ms, +36 ms, −36 ms, +92 ms, −112 ms.

Figure 3 displays the confidence intervals of the AEP amplitude for each of the 18 ms time windows for the left and right temporal site (Figure 3A) for the monolingual vs. bilingual group (Figure 3B), and each group and site by stimulus for the three target time windows (Early: 86–104 ms, Mid 1:146–164 ms, Mid 2: 166–184 ms; Figure 3C).

### 4.1 Early time interval (86–104 ms)

Table 3 displays the results of the linear mixed effects model for the Early time window. There were no significant effects of Group [$\chi_{\left(1\right)}^{2}$ = 0.13, *p* > 0.05] or Stimulus [$\chi_{\left(4\right)}^{2}$ = 1.89, *p* > 0.05]. The two-way interactions of Group by Stimulus and Stimulus by Site were not significant [$\chi_{\left(4\right)}^{2}$ = 0.73, *p* > 0.05, $\chi_{\left(4\right)}^{2}$ = 3.25, *p* > 0.05, respectively]. However, the effect of Site was significant [$\chi_{\left(1\right)}^{2}$ = 5.83, *p* = 0.016]. Specifically, the amplitude in the Early interval was more negative for T7 than T8 (Figure 3A). An interaction between Group and Site was marginally significant [$\chi_{\left(1\right)}^{2}$ = 3.57, *p* = 0.059]. As seen in Figure 3B, the effect of Site tended to be larger for the bilingual than monolingual group. The three-way interaction of Group, Stimulus, and Site was significant [$\chi_{\left(4\right)}^{2}$ = 10.09, *p* = 0.039]. As displayed in Figure 3C, the hemisphere effect on the five stimuli differed for the monolingual and bilingual group.

**Table 3:** Main analysis for the Early time interval: analysis of deviance table (Type III Wald chi-square tests).

**Fixed factor**	**Chisq**	**Df**	***p***	
(Intercept)	22.90	1	< 0.001	^***^
Group	0.13	1	>0.05	
Stimulus	1.89	4	>0.05	
Site	5.83	1	0.016	^*^
Group: Stimulus	0.73	4	>0.05	
Group: Site	3.57	1	0.059	^.^
Stimulus: Site	3.25	4	>0.05	
Group: Stimulus: Site	10.09	4	0.039	^*^

^.^*p* < 0.10; ^*^p < 0.05; ^***^p < 0.001.

*Post-hoc* analyses following up the three-way interaction revealed the following pattern: the bilingual group showed a significant difference between T7 and T8 for the +36 ms VOT stimulus (β = −1.56, *SE* = 0.66, *t* = −2.35, *p* = 0.020) and for the −36 ms VOT stimulus (β = −1.77, *SE* = 0.66, *t* = −2.67, *p* < 0.01), where the right site (T8) was more positive than the left (T7). The monolingual group showed a significant difference between T7 and T8 only for the −112 ms VOT stimulus (β = −2.15, *SE* = 0.78, *t* = −2.76, *p* < 0.01), with the right site (T8) being more positive than the left (T7). *Post-hoc* comparisons between the bilingual and monolingual group for each site and stimulus showed a marginally significant difference between monolinguals and bilinguals for the +36 ms VOT stimulus at T8 (β = −1.46, *SE* = 0.87, *t* = −1.67, *p* = 0.097), with the bilinguals showing a more positive response.

Further analyses were conducted to examine whether the relative amount of input in German vs. Italian affected bilingual children's neural response in the Early time interval. A linear mixed effects model was constructed for the bilingual group including fixed effects of relative amount of German input (scaled to center around 0), Stimulus, Site, and all their two-way and three-way interactions. The random effect was by-participant intercepts.

The results demonstrated that there was a significant two-way interaction of German input and Site [$\chi_{\left(1\right)}^{2}$ = 10.50, *p* < 0.01]. Italian-German bilingual speakers who had more German input had a larger hemisphere effect. A Pearson's product-moment correlation test demonstrated that there was a significant positive correlation between the amplitude at the T8 site and the German input, *r*_(105)_ = 0.30, *p* < 0.01. Specifically, the Italian-German bilinguals who had more German input showed more positive (i.e., less negative) Na response across the five stimuli.

### 4.2 Mid-time 1 (146–164 ms) and Mid-time 2 (166–184 ms) interval

Table 4 displays the results of the linear mixed effects model for the Mid 1 and 2 interval. There was a significant effect of Site [$\chi_{\left(1\right)}^{2}$ = 199.61, *p* < 0.001] and Stimulus [$\chi_{\left(4\right)}^{2}$ = 97.16, *p* < 0.001], but no significant effect of Group [$\chi_{\left(1\right)}^{2}$ = 0.07, *p* > 0.05] or Time interval [$\chi_{\left(1\right)}^{2}$ = 0.05, *p* > 0.05]. Generally, the right site (T8) was more positive than the left site (T7) (Figure 3A).

**Table 4:** Main analysis for the Mid 1 and 2 time intervals: analysis of deviance table (Type III Wald chi-square tests).

**Fixed factor**	**Chisq**	**Df**	***p***	
(Intercept)	3.92	1	0.048	^*^
Group	0.07	1	>0.05	
Time interval	0.05	1	>0.05	
Stimulus	97.16	4	< 0.001	^***^
Site	199.61	1	< 0.001	^***^
Group: Time interval	0.26	1	>0.05	
Group: Stimulus	6.24	4	>0.05	
Time interval: Stimulus	10.53	4	0.032	^*^
Group: Site	8.30	1	< 0.01	^**^
Time interval: Site	0.73	1	>0.05	
Stimulus: Site	21.95	4	< 0.001	^***^
Group: Stimulus: Site	9.19	4	0.057	^.^

^.^*p* < 0.10; ^*^p < 0.05; ^**^p < 0.01; ^***^p < 0.001.

There was also a significant two-way interaction of Group and Site, suggesting that the Site effect (T7 vs. T8) was larger for the monolingual than the bilingual group [$\chi_{\left(1\right)}^{2}$ = 8.30, *p* < 0.01]. Figure 3B shows that the amplitude difference for the left vs. right site was larger for monolingual than for bilingual participants. This pattern emerges because the monolingual group tended to show greater positivity at T8 and greater negativity at T7 compared to the bilinguals. There was also a significant interaction of Time interval and Stimulus [$\chi_{\left(4\right)}^{2}$ = 10.53, *p* = 0.032]. Specifically, the effect of Time interval (Mid 1 vs. Mid 2) was different across stimuli. The *post hoc* analyses to follow up this interaction revealed a significant difference between the Mid 1 and Mid 2 interval only for the −112 ms VOT (β = 0.90, *SE* = 0.35, *t* = 2.55, *p* = 0.011), where the Mid 2 interval was generally more negative than the Mid 1 interval.

A significant interaction of Stimulus and Site was found [$\chi_{\left(4\right)}^{2}$ = 21.95, *p* < 0.001]. The *post hoc* analyses revealed the following patterns, summarized in Tables 5A, 5B. At the right site (T8), both the +92 ms VOT and the −112 ms VOT stimulus were more negative than the three shorter VOT stimuli, 0 ms, +36 ms and −36 ms. At the left site (T7), the −112 ms VOT stimulus was more negative than the three shorter VOT stimuli (0 ms, +36 ms, and −36 ms), but also more negative than the +92 ms VOT stimulus. In addition, at the left site (T7), the +92 ms VOT stimulus was more negative than the 0 ms VOT stimulus but did not differ from the −36 ms or +36 ms VOT stimulus.

**Table 5A:** *Post-hoc* analyses for the Stimulus effect of T7 (Left hemisphere).

**Contrasts**	**Estimate**	***SE***	***t***	***p***	
VOT 0 ms vs. VOT +36 ms	0.70	0.36	1.97	>0.05	
VOT 0 ms vs. VOT −36 ms	0.62	0.36	1.74	>0.05	
VOT 0 ms vs. VOT +92 ms	1.01	0.35	2.85	0.036	^*^
VOT 0 ms vs. VOT −112 ms	2.65	0.35	7.52	< 0.001	^***^
VOT+36ms vs. VOT − 36 ms	−0.08	0.36	−0.22	>0.05	
VOT +36 ms vs. VOT +92 ms	0.30	0.36	0.85	>0.05	
VOT +36 ms vs. VOT −112 ms	1.95	0.36	5.41	< 0.001	^***^
VOT −36 ms vs. VOT +92 ms	0.38	0.36	1.07	>0.05	
VOT −36 ms vs. VOT −112 ms	2.03	0.36	5.64	< 0.001	^***^
VOT +92 ms vs. VOT −112 ms	1.64	0.36	4.63	< 0.001	^***^

All *p*-values were adjusted with the Tukey method. ^*^p < 0.05; ^***^p < 0.001.

**Table 5B:** *Post-hoc* analyses for the Stimulus effect of T8 (Right hemisphere).

**Contrasts**	**Estimate**	***SE***	***t***	***p***	
VOT 0 ms vs. VOT +36 ms	0.25	0.36	0.71	>0.05	
VOT 0 ms vs. VOT −36 ms	−0.42	0.36	−1.19	>0.05	
VOT 0 ms vs. VOT +92 ms	1.83	0.35	5.18	< 0.001	^***^
VOT 0 ms vs. VOT −112 ms	1.40	0.35	3.99	< 0.001	^***^
VOT +36 ms vs. VOT −36 ms	−0.68	0.36	−1.87	>0.05	
VOT +36 ms vs. VOT +92 ms	1.57	0.36	4.37	< 0.001	^***^
VOT+36ms vs. VOT − 112 ms	1.15	0.36	3.19	0.013	^*^
VOT −36 ms vs. VOT +92 ms	2.25	0.36	6.25	< 0.001	^***^
VOT −36 ms vs. VOT −112 ms	1.83	0.36	5.07	< 0.001	^***^
VOT +92 ms vs. VOT −112 ms	−0.42	0.36	−1.19	>0.05	

All *p*-values were adjusted with the Tukey method. ^*^p < 0.05; ^***^p < 0.001.

There was no interaction between Group and Time interval [$\chi_{\left(1\right)}^{2}$ = 0.26, *p* > 0.05] or Group and Stimulus [$\chi_{\left(4\right)}^{2}$ = 6.24, *p* > 0.05]. The 3-way interaction of Group, Stimulus, and Interval was excluded because it did not improve the model fit. Specifically, the effect of the two intervals on the five stimuli did not differ between the monolingual and the bilingual group.

The three-way interaction of Group, Stimulus, and Site was marginally significant, $\chi_{\left(4\right)}^{2}$ = 9.19, *p* = 0.057 (Figure 3C). The *post hoc* analyses demonstrated that there was a significant difference in amplitude between the monolingual and the bilingual group for the 0 ms VOT stimulus at the right site (T8) (β = 1.39, *SE* = 0.69, *t* = 2.01, *p* = 0.046), but not for any of the other stimuli at either T7 or T8, *p* > 0.05. Specifically, the bilingual group showed a more negative response to the 0 ms VOT stimulus than the monolinguals.

Further analyses were conducted for the Mid 1 and Mid 2 Time interval to test whether the amount of input in German vs. Italian affected bilingual children's neural responses. To this end, a linear mixed effects model was constructed for the bilingual group with fixed effects of the amount of German input (scaled to center around 0), Stimulus, Site, and Time interval. In addition, six two-way interactions (German input & Stimulus, German input & Site, Stimulus & Site, German input & Time interval, Stimulus & Time interval, and Site & Time interval), and a three-way interaction (German input, Stimulus & Site) were included. By-participant random intercepts were also included.

The results demonstrated that there was a significant two-way interaction of German input and Site [$\chi_{\left(1\right)}^{2}$ = 10.79, *p* < 0.01]. Specifically, the Italian-German bilingual children who had more German input showed a larger difference between T7 and T8 amplitude. A Pearson's product-moment correlation test demonstrated that there was a significant positive correlation between the amplitude at the right site (T8) and German input, *r*_(212)_ = 0.21, *p* < 0.01. That is, Italian-German bilinguals who had more German input showed a more positive amplitude at T8 across all five stimuli for the Mid 1 and Mid 2 interval.

## 5 Discussion

Our findings suggest that the effect of language experience on neural encoding of speech information is complex. The current study observed no main effects of language group, but there were significant interactions of group with site and stimulus. We replicated the finding of a more negative response in the Ta-Tb latency range for bilingual compared to monolingual participants, specifically for the 0 ms VOT stimulus at the right site (T8). We also observed group differences in the early time range where the Na is prominent, which we did not predict. The group differences at the right site (T8) for the +36 ms VOT stimulus revealed a tendency for the bilinguals to have a more positive Na than the monolinguals. Examining this further in terms of amount of input, revealed that bilinguals with more German input had more positive Na amplitudes, as well as a more positive amplitude in the Ta-Tb time interval.

### 5.1 Delayed neural commitment

We suggested that the more negative Ta amplitude for bilinguals observed in the previous studies with Turkish-German and Spanish-English bilinguals compared to monolinguals might be due to a delay in committing to the phonetic patterns of a native language (Rinker et al., 2017, 2022). In the current study, this was clearly seen for the 0 ms VOT stimulus, where bilinguals had a more negative response at T8 for the time intervals where Ta and Tb were expected. The 0 ms VOT stimulus can be considered the most similar to the /ε/ vowel used in our previous studies because it consists of a burst followed immediately by vowel transition (Rinker et al., 2017, 2022). The group by site interaction also confirmed this pattern more generally, with the monolingual children tending to show a more positive response at T8 across all stimuli in the Mid 1 and Mid 2 time interval, where Ta and Tb are expected. We will address the question of whether Tb is present in Section 5.3.

We argue that the attenuation of positivity of Ta is more consistent with the hypothesis that bilingual children show delayed commitment than the alternative hypothesis, which is that differences are related to whether the speech sound is a close match to a native language category. First, the 0 ms VOT stimulus clearly fell within the German /ba/ or the Italian /pa/ phoneme category. Thus, all the children in the study, whether monolingual or bilingual must have had considerable experience with the acoustic-phonetic correlates of the short-lag VOT. Second, we did not find clear effects of differences in processing the prevoiced stimuli between the German monolinguals and the Italian-German bilinguals. The expectation was that the monolingual German group would have had little to no experience with the prevoiced category. However, Hamann and Seinhorst (2016) found that some German speakers—similar to English speakers (see Davidson, 2016)—may also prevoice short-lag stops, but in the context of the current study, where the ISI between stimuli was a minimum of 300 ms (depending on the stimulus), prevoicing would not be expected. Thus, we argue that if the delayed emergence of Ta was simply due to experience with the acoustic-phonetic correlates of a speech sound, then we would have seen a more negative Ta response for the German monolingual compared to the Italian-German bilingual group for the prevoiced stimuli and no group differences for the short-lag or the long-lag VOT stimuli.

Our previous experiments with children examined only one token of one vowel phoneme, and thus, we were not able to distinguish between the two explanations. We know of only one other study that has examined the T-complex in bilingual listeners and it is with adults (Wagner et al., 2013). They found that bilingual Polish-English listeners showed a more negative Ta to the syllable onset /pət/ than /pt/, whereas English listeners showed no difference. The /pt/ onset is a phonotactic violation in English. The pattern of findings for the T-complex may be different for adults because they have already fully committed to their speech-sound categories. It is also possible that the pattern is different when listeners are asked to perform a task with the stimuli. In the Wagner et al. (2013) study, participants were asked to judge whether the second word of a word pair was two or three syllables (e.g., /pətola/-/ptola/, where the second word is two syllables). It will be important to follow bilingual participants longitudinally to determine how encoding of speech, as indexed by the T-complex, develops in relation to their experience with their two languages.

### 5.2 Na peak

We did not expect to find group differences between the monolingual and bilingual children for the Na time interval. Na is arguably the opposite pole of the P1 dipole and reflects processing in superior temporal cortex (Tonnquist-Uhlén et al., 2003). To date, little evidence indicates that language experience influences the neural sources underlying P1. The *post-hoc* tests did not reveal direct differences between monolinguals and bilinguals for the Na. Rather, the left vs. right site showed differences between the two groups for some stimuli. The pattern for these differences, however, does not lead to a coherent explanation. The bilinguals showed hemisphere differences for the +/– 36 ms VOT stimuli, whereas the monolinguals showed a difference only for the −112 ms VOT stimulus. The finding that bilingual listeners with more German input showed the more positive amplitude for Na also appears to be counter intuitive. However, a possible explanation is that this effect is actually attentional. Specifically, a previous study revealed that greater attention to speech can lead to a negative shift of the P1-N1-P2 response at superior sites (Datta et al., 2021). It is possible that this pattern inverts at temporal sites. Under this explanation, the bilingual participants with more German input were allocating more attention to the speech stimuli. Replication of these findings for Na will be necessary, as well as direct manipulation of attention to have confidence that this pattern of findings is related to language experience rather than to some other factor, or simply to noise in the data and to provide support for the suggestion that attention shifts to the speech signal might account for this effect on Na.

The Na peaks to the CV syllables in the current study appear later than what has been found for vowels and tones in previous papers (e.g., Tonnquist-Uhlén et al., 2003; Bishop et al., 2011), but this might be related to the complex nature of the CV stimuli, which included a transition from the onset of voicing into the more steady-state vowel. In addition, the long-lag +92 ms VOT stimulus and the prevoiced −112 ms VOT stimulus both showed a second negative peak with a timing that is consistent with Na elicited to the onset of the vowel. This second “Na” overlaps with the Ta-Tb time window and will be further discussed in the next section.

### 5.3 Ta vs. Tb

There was no evidence of a negativity consistent with the Tb for the 0 ms, +36 ms or −36 ms VOT stimulus. Specifically, the raw data waveforms showed no clear Tb peak following the Ta peak. The response to the +92 ms and −112 ms VOT stimuli showed negativity in the Tb time window, but, as we point out above, this negative deflection is likely to include Na to the vowel onset. That is, Na to the vowel summates with Tb to lead to a clear negative peak. Previous studies indicated that a Tb is identifiable in 5-year-old children, to a range of stimuli (tone, speech sounds, and clicks) (Tonnquist-Uhlén et al., 2003; Rinker et al., 2017, 2022) and that it increases in amplitude with age (Tonnquist-Uhlén et al., 2003). Two papers that include children younger than 5 years of age suggest that Tb emerges between 4 and 5 years of age (Rinker et al., 2017; Shafer et al., 2015). Specifically, in monolingual children, a reliably identifiable Tb peak was observed in at least 60% of children over 4 years of age (Rinker et al., 2017; Shafer et al., 2015). For bilingual Spanish-English and German-Turkish children, however, the percentage of participants showing a clear Tb peak was lower than for monolinguals (Rinker et al., 2017). This lack of distinction could be due to either an attenuated Ta (less positive) or attenuated Tb (less negative).

In the current study, we included time intervals in the analysis for the 146–184 time range because we expected the early time interval to be more positive, reflecting Ta and the later time interval to be more negative, reflecting Tb. We did observe a stimulus by time interval effect, but *post-hoc* tests revealed that time interval was significant only for the −112 ms VOT stimulus. As already noted, we suggest that the increased negativity of the later time interval is likely to reflect the Na response to the vowel onset. The lack of a clear Tb could be due to age and/or stimulus factors. The mean age of the children in the current study was about 5 years of age (59 months for monolinguals and 61 months for bilinguals), ranging from 47 to 73 months, which is younger than the German and Turkish-German children in Rinker et al. (2017, 2022), but which matches the age range of the New York City sample (English monolingual and Spanish-English bilingual) in Rinker et al. (2017). In the younger sample (4 to 5 years), more children were missing the Tb peak. Specifically, only 65% of the monolinguals and 61% of the bilinguals showed Tb at T8, and only 65% of monolinguals and 44% of bilinguals showed Tb at T7. In addition, the VOT stimuli in the current study were longer and more complex than those in previous studies with children (i.e., V vs. CV stimuli). The phonetic properties of these VOT stimuli would result in Na, Ta, and Tb to the onset of the voicing and to the onset of the vowel overlapping in a manner to cancel out the Tb effect for some stimuli. In the current study, identifying a clear Tb peak in the individual data was challenging both because children often lacked a deflection and because the stimulus difference of when the vowel started in relation to the onset of phonetic information led to uncertainty about whether a negativity was Tb to the stimulus onset or Na to the vowel onset. The data from the current study provide insight into this relationship. However, it will be necessary to examine T-complex measures to these stimuli in a mature population to further determine how these components summate.

The second Na to the vowel onset can also be viewed as indication of an acoustic change. Studies designed to examine the acoustic change (specifically, the acoustic-change complex or ACC) typically focus on P1-N1-P2 at frontocentral sites (Martin et al., 2010). The obligatory response to the stimulus onset is usually the strongest, with attenuation to a following stimulus change when the ISI is brief. The Na negativity to the vowel onset in the current study may be quite large because the acoustic energy in the prevoiced and long-lag stimuli was quite weak compared to that of the vowel. Further studies need to be undertaken to explore how the acoustic correlates of various complex syllable shapes influence the AEP morphology. In addition, developmental studies are needed to determine when Tb emerges to these complex speech shapes.

### 5.4 Maturation of the T-complex

Several maturational studies of the lateral temporal measures show that Na, Ta, and Tb are identifiable in individual data by 5 years of age (Tonnquist-Uhlén et al., 2003; Shafer et al., 2015). The latency of the Ta and Tb peaks were shown to shift less across age than obligatory responses P1, N1, and P2 at superior sites, leading to the suggestion that the lateral cortex sources underlying these peaks mature earlier than those in the superior temporal cortex (Tonnquist-Uhlén et al., 2003). In addition, the Na latency correlated significantly, but weakly with P1 at T8 in Tonnquist-Uhlén et al. (2003), whereas correlations between Ta and Tb and N1b and P2, respectively were non-significant. This pattern of findings suggests that Ta and Tb are independent of the sources underlying N1b and P2. Tonnquist-Uhlén et al. (2003) argue that the T-complex Ta and Tb mature earlier than the P1-N1b-P2 complex. This is particularly interesting considering the finding of amplitude differences between the monolingual and bilingual group in time intervals for the Ta and Tb. There is no reason to believe that bilingual children have a less mature lateral temporal cortex than monolingual children. We therefore maintain that the better explanation is that bilingual children are delaying neural commitment to speech information because they need additional input in the two languages before the neural sensitivity to speech sounds declines (Johnson and Newport, 1989; Birdsong and Molis, 2001; Hartshorne et al., 2018) although longitudinal investigations will be necessary to substantiate this claim.

### 5.5 Role of input

Our previous study (Rinker et al., 2017) showed a complex relationship between amount of input and Ta positivity. Specifically, for the Turkish-German children, more input in German led to a more positive Ta. The finding for the Spanish-English children was different, but probably because these children all had considerable English input (ranging from balanced Spanish and English to dominant English). For the children who were clearly dominant in English (with weak Spanish skills), more English led to more positive responses. But for those who had strong Spanish skills, those who were the most balanced showed the most positive responses. Therefore, in the current study, input was not only used as a binary measure (cf. language status, i.e., being bilingual vs. monolingual) but also as a continuous variable reflecting the relative amount of exposure to their two languages. In fact, the amount of input in Italian vs. German varied greatly across our bilingual participants. In the current study, bilingual children with more input in German showed a more positive amplitude in the Ta-Tb time window, which more closely resembled the monolingual German pattern. Future studies will be needed to identify how much input in a second language will lead to modulation of how speech information is encoded in the lateral temporal cortex and the time course of maturation of the T-complex under different input conditions, as well as the relationship of neural processing to behavioral perception.

### 5.6 Clinical implications

One challenge of assessing multilingual children for developmental language disorder (DLD) is the considerable variability in the development of the two (or more) languages. Many researchers have attempted to find neural biomarkers for DLD, but success has been elusive. Several previous studies have observed attenuated Ta and/or Tb to auditory information in children with developmental language delays (e.g., Tonnquist-Uhlén, 1996; Shafer et al., 2011b; Bishop et al., 2012; Rinker et al., 2022). The poor responses at temporal sites were argued to result from poor auditory processing. Another possibility is that children with DLD have delayed maturation of auditory cortex (McArthur and Bishop, 2004). However, the finding of attenuated Ta and Tb for both typically developing bilingual children and children with DLD undermines the use of Ta/Tb as a biomarker. Several studies have also observed that children with DLD show attenuation of frontocentral P1-N1b-P2 responses (Bishop and McArthur, 2004; Tonnquist-Uhlén et al., 1996). If children with DLD show attenuation of both P1-N1b-P2 and T-complex responses, whereas children with bilingual input only show attenuation of T-complex responses, then the T-complex data will still serve to provide insight on DLD when used in combination with the frontocentral measures. More specifically, it will be important to explore whether a possible neural pattern for monolingual children with DLD is robust P1-N1-P2 and attenuated T-complex to speech sounds and if this pattern exists, then to explain how this pattern relates to DLD. We hypothesize that monolingual children with DLD will show poor neural encoding and processing at both frontocentral and temporal sites, which will distinguish them from children with typical language skills who are acquiring two or more languages.

### 5.7 Theoretical implications

A continuing debate in linguistic theory has focused on the abstractness of phonological categories (Calabrese, 2012). Evidence from neural studies has revealed that speech information is represented in the brain at the level of the obligatory P1-N1-P2 complex with considerable detail (veridical; Breen et al., 2013). At higher levels, such as neural processing indexed by the MMN, however, phonological status modulates the responses. The findings of our study indicate that neural processing indexed by the T-complex is also modulated by phonological status. It will be of considerable interest to further explore the maturation of the neural mechanism underlying these three measures (P1-N1-P2, T-complex and MMN) in relation to amount of input and use of two (or more) languages from the preschool years up to adulthood.

## 6 Conclusion

This study suggests that bilingual experience generally affects encoding of speech sounds in lateral cortex, rather than affecting only phonetic patterns from the weaker (less input) language. We argue that these findings support the hypothesis that bilingual children delay neural commitment to the language-specific phonetic detail of both their languages. This delay in commitment is likely to be beneficial to bilingual children in that it allows them more time to establish the speech perception routines that will be most efficient for communication in the child's two languages. We suggest that this delay is most apparent for children with more balanced input in the two languages because children who were dominant in German more closely resembled the monolingual German children. To further test this hypothesis, it will be important to follow bilingual/multilingual children's development from 4 years of age through puberty to determine when neural commitment occurs and how speech encoding and processing is modulated by fluctuations in the amount of input in a bilingual/multilingual child's languages.

## Data Availability

The raw data supporting the conclusions of this article will be made available by the authors, without undue reservation.

## Footnotes

^1^Psychology Software Tools Inc. [E-Prime 3.0]. (2016). Available at: https://support.pstnet.com/.
